# Current State of Personalized Genitourinary Cancer Radiotherapy in the Era of Precision Medicine

**DOI:** 10.3389/fonc.2021.675311

**Published:** 2021-05-07

**Authors:** Sophia C. Kamran, Jason A. Efstathiou

**Affiliations:** Department of Radiation Oncology, Massachusetts General Hospital, Harvard Medical School, Boston, MA, United States

**Keywords:** genitourinary cancer (GU cancer), personalized radiation oncology, precision oncology, prostate cancer, testicular cancer, bladder cancer, renal cell carcinoma, precision medicine

## Abstract

Radiation therapy plays a crucial role for the management of genitourinary malignancies, with technological advancements that have led to improvements in outcomes and decrease in treatment toxicities. However, better risk-stratification and identification of patients for appropriate treatments is necessary. Recent advancements in imaging and novel genomic techniques can provide additional individualized tumor and patient information to further inform and guide treatment decisions for genitourinary cancer patients. In addition, the development and use of targeted molecular therapies based on tumor biology can result in individualized treatment recommendations. In this review, we discuss the advances in precision oncology techniques along with current applications for personalized genitourinary cancer management. We also highlight the opportunities and challenges when applying precision medicine principles to the field of radiation oncology. The identification, development and validation of biomarkers has the potential to personalize radiation therapy for genitourinary malignancies so that we may improve treatment outcomes, decrease radiation-specific toxicities, and lead to better long-term quality of life for GU cancer survivors.

## Introduction

Radiation therapy is instrumental in the management of many genitourinary malignancies. Technological advances in imaging, treatment planning, and treatment delivery have allowed physicians to deliver higher radiation dose to tumor or tumor bed while minimizing dose to surrounding normal tissue. Other advances in screening and other treatment options have translated to improvements in clinical outcomes for patients with genitourinary malignancies. Yet, the optimal management for malignancies on an individualized level is not well understood. There is an urgent need to incorporate more biomarkers to personalize radiation therapy in the era of precision oncology. Here, we review the progress in the identification, development, and validation of biomarkers for genitourinary malignancies to guide treatment recommendations, as well as highlight challenges and opportunities for further investigations in personalized radiation medicine.

## Prostate Cancer

Prostate cancer screening, risk stratification, and treatment have advanced dramatically in the past decades. Despite this, the optimal combination of treatments for an individual is not clear nor personalized at this time. The ideal management for an individual with prostate cancer is a complicated decision process, with more than one approach often available. It is therefore imperative to determine which patients are more likely to benefit from a treatment over another, both in terms of cancer control and quality of life, in keeping with precision medicine principles. In addition, it is critical to improve diagnosis and risk stratification with respect to detecting both clinically significant and biologically aggressive disease. Below, we briefly review the current state of various innovative predictive/prognostic tools from detection through risk stratification, as well as advances in radiation delivery to further target the prostate tumor biology.

### Detection and Screening

Prostate cancer detection has been aided by the use of magnetic resonance imaging (MRI). Early use in the 1990s allowed clinicians to evaluate for high-risk features such as extracapsular extension and seminal vesicle invasion (1, 2). Technology evolved and the inclusion of multiple parameters to evaluate the prostate, also known as the multiparametric MRI (mpMRI), has allowed for accurate localization of suspected prostate cancer lesions (3–6). At this time, tissue diagnosis with biopsy remains the gold standard, however mpMRI is a robust supplement in the diagnosis of prostate cancer. In addition, mpMRI has been increasingly incorporated into prostate biopsies by serving as a guide for “targeted” lesions that are not a part of the standard systematic biopsies. This can be accomplished using either a “cognitive” fusion biopsy or an MRI-transrectal ultrasound fusion biopsy. Both have demonstrated improved detection of clinically significant disease and overall disease burden in multiple studies (7–11). A recent phase 3 randomized trial determined that an MRI followed by selected targeted biopsy was noninferior to a standard 12-core transrectal ultrasound biopsy in detecting Gleason 3 + 4 (Grade Group 2) disease or higher (12). mpMRI will continue to play a large role in the detection of prostate cancer as well as in active surveillance.

There are multiple biomarkers that exist designed to be used to aid in the diagnosis of prostate cancer before a positive prostate biopsy (**Table 1**). Serum biomarkers notably include: 1) the Prostate Health Index (PHI) which combines total prostate-specific antigen (PSA), free non-protein bound PSA (fPSA), and an isoform of fPSA known as p2PSA (13); and 2) the 4Kscore, which incorporates serum biomarkers including total PSA, fPSA, intact PSA, and human kallikrein 2, as well as clinical variables to predict risk of high-grade PCa on the biopsy (14). Notable urinary biomarkers include prostate cancer antigen 3 (PCA3), which is a noncoding messenger RNA (mRNA) overexpressed in prostate cancer tissue and detectable in urine after a digital rectal examination (DRE) (15). The TMPRSS2-ERG genomic rearrangement can be detected in post-DRE urine samples with a specificity of 93% and a positive predictive value of 94% for prostate cancer diagnosis (16). TMPRSS2 is an androgen-regulated gene. ERG is a transcription factor that is overexpressed in ~50% of primary prostate tumors (17). TMPRSS2-ERG fusions are described in 30-50% of new prostate cancer diagnoses (18). There are multiple other biomarkers as per **Table 1** that can aid in the diagnosis of prostate cancer and have been shown to outperform PSA as a diagnostic tool, however their use in clinical practice is variable due to their limitations. There is a need to validate and compare these biomarkers against each other in a prospective manner before incorporating into routine clinical practice.

**Table 1:** Biomarkers for prostate cancer screening.

Test	Biomarker	Positive
*Blood-based*		
4K	Total PSA, fPSA, intact PSA, human kallikrein 2 as well as clinical variables (age, DRE, and prior biopsy results)	≥9%
Prostate Health Index	Total PSA, fPSA (free non-protein bound PSA), and p2PSA (isoform of fPSA) Formula: (2pPSA/fPSA)x√PSA	≥25
*Urine- post DRE*		
PCA3	Concentration of PCA3 mRNA relative to PSA mRNA	≥35
TMPRSS2-ERG	Detection of the fusion gene in post-DRE urine	≥10
MiPS (PCA3) + TMPRSS2-ERG	Combination of PCA and TMPRSS2-ERG	≥35 + ≥10
SelectMDX	RNA levels of *DLX1* and *HOXC6* Also includes total PSA, PSA density, DRE, age, family history	≥2.8RS
ExoDx	RNA content in extracellular vesicles, measuring RNA and calculating as sum of normalized RCA and RNA ERG	≥15,6

DRE, digital rectal examination.

Molecular imaging, most notably, has exploded on the scene in the past few years with the development of several radiolabels specific for prostate cancer. Historically, the role of PET/CT was limited for prostate cancer diagnosis/staging. Multiple PET imaging tracers are being evaluated, with the top three most explored/promising summarized in **Table 2**. ^11^C-choline is a radiotracer that was previously explored for prostate cancer diagnosis, however its sensitivity and specificity values ranged from 72-87% and 62-84% respectively. In addition, choline-avid PET images could not reliably distinguish between benign and malignant lesions (19). Since that time, the PET compound ^18^F-fluciclovine demonstrated promise for prostate cancer detection, particularly in the setting of biochemically recurrent prostate cancer (20). Generally, the sensitivity improves with a higher PSA relapse level. Due to its improved technique to detect prostate cancer lesions at lower PSAs in the recurrent setting compared to conventional imaging, this scan is FDA-approved for use in the post-treatment biochemically recurrent clinical setting. More recently, studies using targeted radiolabeling of the prostate-specific membrane antigen (PSMA) for PET/CT demonstrate promising data (21, 22). PSMA is a transmembrane protein that is overexpressed on prostate cancer tumor tissue; thus this can be targeted with a radioligand and lead to enhanced prostate cancer uptake and detection. Gallium 68 PSMA-11 has recently received FDA approval for use in the United States for PET imaging of PSMA positive lesions in patients with suspected prostate cancer metastasis as well as patients with suspected prostate cancer recurrence based on elevated PSA levels. Broad distribution of Gallium 68 PSMA-11 is being worked on. The proPSMA prospective trial demonstrated the superiority of PSMA PET-CT (n=150 men) compared to conventional imaging (n=152 men) for accuracy of identifying pelvic nodal or distant-metastatic disease, with a 27% absolute greater area under the curve (AUC) for accuracy over conventional imaging (92% [88-95] *vs* 65% [60-69] (23). PSMA PET imaging appears to be better than fluciclovine PET at lower PSA levels. A single institutional comparison between PSMA PET and fluciclovine PET in prostate cancer patients with biochemical recurrence after radical prostatectomy (PSA <2.0 ng/mL) found that, in 50 enrolled patients, detection rates were lower with fluciclovine PET (13 of 50, 26%) versus PSMA PET (28 of 50, 56%), with an odds ratio of 4.8 at the patient level (95% CI 1.6-19.2, p=0.0026) (24).

**Table 2:** Summary of top PET imaging tracers for prostate cancer.

PET tracer	Production method	Half-life
Carbon 11 (^11^C) choline	Cyclotron (onsite)	20.3 min
Gallium 68 (^68^Ga) PSMA	Generator	67.7 min
Fluorine 18 (^18^F) fluciclovine	Cyclotron (regional)	109.8 min

However, not all metastatic disease, particularly castrate-resistant metastatic disease, expresses PSMA. There are many ongoing trials further defining the role of Gallium 68 PSMA-11 PET in both the biochemically recurrent and diagnostic setting, particularly for advanced disease (25).

### Risk Stratification

It has become increasingly evident that using clinicopathologic characteristics alone to stratify patients into various risk categories to guide treatment decisions may be insufficient, as we learn more about heterogenous outcomes in risk groups. There are newer clinical staging/risk-stratification systems that demonstrate promise. These include the STAR-CAP (26) as well as the CAPRA score (27). However, there remains a need to identify and validate markers intrinsic to tumor biology to further stratify patients into risk groups. To that end, there are currently four commercially available gene panels that can be used for localized prostate cancer (**Table 3**). These can be used to further risk-stratify patients to guide personalized treatment decisions.

**Table 3:** Molecular tests for Prostate Cancer Risk Stratification.

Genomic classifier	Test	Test independently predicts
PROLARIS	46 genes (15 housekeeper, 31 cell cycle progression genes) to determine a cell cycle progression score	-Prostate cancer-specific mortality -Biochemical recurrence -Metastases -Grade group ≥3 or pT3 at time of surgery
PROMARK	Expression of 8 genes	-Prostate cancer-specific mortality
ONCOTYPE	17 genes associated with prostate cancer to create Genomic Prostate Score	-Metastases -Prostate cancer-specific mortality -Grade group ≥3 and/or pT3+ disease at time of surgery
DECIPHER	22 RNA biomarkers	-Metastases -Prostate cancer-specific mortality -Postoperative radiation sensitivity -Lymph node metastases - Grade group ≥3 or pT3+ disease at time of surgery -Biochemical failure -Grade group ≥4 at time of surgery

Prolaris (Myriad Genetics, Salt Lake City, UT) is a gene expression panel consisting of 46 genes (15 housekeeper genes, 31 cell cycle progression genes) which results in a cell cycle progression (CCP) score. It is designed for use on biopsy, transurethral resection of the prostate (TURP) specimens, as well as radical prostatectomy specimens. There are a few studies that have evaluated the utility of this biomarker for clinical decision-making. Cuzick et al. used Prolaris on biopsy specimens and determined that the CCP score was the strongest independent predictor of death (28); separately the same group evaluated Prolaris in both prostatectomy and TURP specimens, demonstrating its strong performance as a prognostic factor for biochemical recurrence and time to death, respectively (29). Cooperberg et al. found that the CCP score was able to surpass the performance of a standard postoperative risk assessment score and had improved accuracy of risk stratification for outcomes for men with localized prostate cancer (30). Freedland et al. validated the Prolaris CCP score in the context of men receiving external beam radiotherapy, demonstrating its superior performance to predict recurrence and was associated with prostate cancer-specific mortality (31). Finally, a critical assessment of Prolaris by NICE determined that the use of Prolaris changed clinicians’ treatment decisions in at least 47% of cases (32).

The Promark assay (Metamark Genetics Inc, Waltham, MA) uses the expression of eight different genes. This assay was validated using intact tissue biopsies and aids in classification for non-favorable pathology, providing independent prognostic data for stratifying patients (33).

A separate assay consisting of 17 genes was developed called Oncotype DX Genomic Prostate Score (Genomic Health, Redwood City, CA). The expression of these genes is incorporated into an algorithm to create a Genomic Prostate Score (GPS). The score was demonstrated to improve prediction of presence of adverse pathology (34, 35). Interestingly the first prospective study evaluating GPS after initial active surveillance found that there was no association of GPS with adverse pathology in those who underwent radical prostatectomy. There was also no association with upgrading in the surveillance biopsy (36).

The last assay to mention is the Decipher genome classifier (GenomeDx Biosciences, Vancouver, BC, Canada). This assay is based on the analysis of the expression of 22 genes. Decipher has been validated in multiple studies (37–41). A separate study found that in men who underwent radical prostatectomy followed by radiation, Decipher predicted both metastasis and biochemical recurrence (38). A meta-analysis published in 2017 confirmed the prognostic value of the Decipher score, independent from clinicopathologic variables. The meta-analysis included data from five studies in men who underwent radical prostatectomy. A low Decipher score was found to be associated with long-term disease control after surgery, while a higher score was found to be associated with a worse prognosis (41). There are multiple additional studies confirming the utility of Decipher in the post-operative setting, thus this is the assay with the strongest evidence to date. In addition, the Decipher test has been used in the intact setting. In a retrospective multicenter cohort study of 266 with very low, low, and favorable-intermediate risk men, it was found that the Decipher score was an independent predictor of adverse pathology, thus it is an aid to appropriately identify good candidates for active surveillance (42). In a cohort of men with intermediate-risk prostate cancer treated with radiation therapy alone, the Decipher score accurately predicted disease recurrence in these individuals at 5 years (area under the curve 0.78, 95% CI 0.59-0.91) (43). Decipher has been validated as part of a clinical-genomic risk group classification for localized prostate cancer to improve risk stratification, finding that 67% of patients would be reclassified from the standard NCCN risk-system by the new system (44). In an ancillary study of the NRG/RTOG 9601 trial, Decipher was validated and independently associated with distant metastases (hazard ratio [HR] 1.17, 95% CI 1.05-1.32, p=0.006), prostate cancer-specific mortality (HR 1.39, 95% CI 1.20-1.63, p<0.001), and overall survival (HR 1.17, 95%CI 1.06-1.29, p=0.002), after adjusting for age, race/ethnicity, Gleason score, T stage, margin status, entry PSA, and treatment arm (45). Based on this data, several trials are incorporating this risk classifier for stratification to either intensification/de-intensification treatment based on either high or low genomic risk, respectively (NRG-GU009, NCT0451371).

For advanced prostate cancer, studies have found that there are multiple mutations present in genes involved in the DNA repair pathways (DDR genes), particularly in patients with metastatic castration-resistant prostate cancer (mCRPC) (46–50). These mutations have been identified as a biomarker of response to poly ADP ribose polymerase (PARP) inhibitors and platinum chemotherapy. The PROfound trial was a randomized phase 3 trial evaluating PARP inhibitor olaparib in men with mCRPC with disease progression (51). Men had to have an alteration in prespecified genes with a direct or indirect role in homologous recombination repair and were divided into cohort A (at least one alteration in *BRCA1, BRCA2*, or *ATM*, n=245 patients) and cohort B (alterations in any of 12 other prespecified genes, n=142 patients). Imaging-based progression-free survival was longer in the olaparib group compared to control in cohort A (7.4 months versus 3.6 months, HR 0.35, 95% CI 0.25-0.47, p<0.001), yet this was less pronounced in cohort B. A significant benefit was found for olaparib in the overall population (cohorts A and B combined). Based on the promising early data (51–58), there are now multiple clinical trials ongoing to evaluate the utility of combining PARP inhibitors and platinum chemotherapy in prostate cancer patients with DDR mutations.

Separately, there have been multiple mutations associated with androgen receptor signaling in aggressive prostate cancer. A study of mCRPC patients with AR amplification who received first-line docetaxel resulted in a lower risk of death for patients with AR amplification, compared to those who received androgen receptor targeting agents (59). Similarly, in men with AR splice variant 7 (AR-S7), studies have found better outcomes with taxane treatment (60–63).

In men with mCRPC, loss of PTEN is common, leading to the overexpression of the PI3K/AKT pathway (47). This has been shown to lead to increased AR signaling and worse overall clinical outcomes (64), thus trials are underway to combine AR-targeted and PI3K/AKT inhibitors in these populations with PTEN loss.

A separate area of great promise for precision medicine and precision ‘omics includes liquid biopsy techniques. This non-invasive technology can allow for biomarker discovery at multiple timepoints without need to rely on biopsies or other means to obtain tissue. Specifically, circulating tumor cells (CTCs), circulating tumor/cell-free DNA (ctDNA, cfDNA) provide snapshots of tumor cells and tumor-derived nucleic acids, respectively. These assays have demonstrated predictive and prognostic promise in metastatic prostate cancer (65) and early data in the localized setting is encouraging (66, 67).

### Advances in Radiotherapy Techniques, Treatment Delivery

Major advances in radiotherapy technique and delivery have led to the ability to target the prostate gland accurately, while largely avoiding normal tissues and sparing toxicity. This has allowed for dose escalation and improved treatment outcomes. Our improved understanding of the radiobiology behind prostate cancer has led to our current efforts and advances in techniques, while eventual integration with genomic tests/molecular understanding of a prostate tumor on an individual level can allow physicians to further personalize radiation therapy with these new techniques.

Innovations in imaging and other technologies have greatly contributed to our ability to “dose-escalate” prostate radiation treatment. For example, the use of a perirectal hydrogel spacer has been shown to be associated with lower dose to the rectum as well as decreased rectal toxicity (68, 69). A multicenter randomized controlled trial demonstrated a reduction in late (defined as 3-15 month) rectal toxicity severity in the spacer group, with 2.0% and 7.0% late rectal toxicity incidence in the spacer and control groups, respectively (p=0.04) (68). This is particularly beneficial for prostate cancer, as multiple hypotheses exist relating to the intrinsic radiobiology of prostate cancer. Emerging evidence suggests that such biology leads to greater sensitivity to increased fraction size (70). Other data suggest that prostate cancer harbors a lower α/β (a metric characterizing tissue/tumor sensitivity to radiation dose per treatment) compared to the surrounding normal tissues. This indicates that hypofractionated radiation (delivery of a higher dose to the prostate gland per treatment, for fewer total treatments) may improve cancer control. Thus, there has been great interest in moderate hypofractionation (generally accepted as 2.4-3.4 Gy per fraction (fx)) as well as ultrahypofractionation (generally accepted as >4-5 Gy/fx) (71, 72). Modern noninferiority trials have demonstrated excellent overall outcomes in comparison to standard fractionation (**Table 4**) (73–76). One superiority randomized trial comparing 75.6 Gy in 1.8 Gy/fx to 72 Gy in 2.4Gy/fx also demonstrated improved cancer control with moderate hypofractionation (77). This approach may be preferred for men with localized prostate cancer given the improved resource utilization and convenience. Ultrahypofractionated is an extreme form of hypofractionation, and there are several ongoing studies exploring its utility for localized prostate cancer. The HYPO-RT-PC trial demonstrated worse acute urinary toxicity with ultrahypofractionation (78). However, in the recently published PACE-B trial, ultrahypofractionation was not found to increase acute genitourinary or gastrointestinal toxicity (79). The ongoing RTOG 0938 trial is a phase II randomized trial evaluating 2 ultrahypofractionation regimens, 36.25 Gy in 5 nonconsecutive fractions or 51.6 Gy in 12 daily fractions; patient-reported outcome data did not demonstrate any significant difference between the two treatment schedules (80).

**Table 4:** Moderate hypofractionation trials for prostate cancer.

Trial	Type	Year	N	Trial arms	Median FU	Primary endpoint	Findings	Toxicities
PROFIT (73)	Noninferiority	2017	1206	78 Gy/39 Fx *vs* 60 Gy/20 Fx	6.0 y	Disease-free survival	HR (95% CI): 0.96 (0.74–1.25)	No significant difference in late toxicity
HYPRO (74)	Noninferiority	2016	804	78 Gy/39 Fx *vs* 64.6 Gy/19 Fx	5.0 y	Relapse-free survival	HR (95% CI): 0.86 (0.63-1.16)	Higher grade 2+ acute GI toxicity with hypoFx; Higher grade 2+ late GU toxicity with hypoFx
CHHiP (75)	Noninferiority	2016	3163	74 Gy/37 Fx *vs* 60 Gy/20 Fx *vs* 57 Gy/19 Fx + 3-6 mo ADT	5.2 y	Time to biochemical failure	HR (95% CI): 0.84 (0.68–1.03) 57 Gy/19 Fx inferior to 74 Gy/37 x	No significant differences but trend toward increased late grade 2+ GU toxicity
RTOG 0415 (76)	Noninferiority	2016	1092	73.8Gy/41 Fx *vs* 70 Gy/28 Fx	5.8 y	Disease-free survival	HR (95% CI): 0.85 (0.64-1.14)	Increased GI/GU late grade 2+ with hypofx
Hoffman et al. (77)	Superiority	2018	206	75.6 Gy/42 Fx *vs* 72 Gy/30 Fx	8.5 y	PSA failure	8-y failure rate 10.7% (95% CI: 5.8%–19.1%) for 72 Gy *vs* 15.4% (95% CI: 9.1%–25.4%) for 75.6 Gy, P = 0.036	Nonsignificant increase in late grade 2+ GI toxicity with hypoFx

A separate method of “dose escalation” involves boosting visible tumor within the prostate that is visualized *via* multiparametric MRI with external beam radiation therapy. A recent phase III randomized controlled trial (FLAME) evaluated the utility of a focal lesion microboost in patients with intermediate- and high-risk prostate cancer (81). This demonstrated improved biochemical disease-free survival in the men who received the focal boost compared to the standard arm (HR 0.45, 95% CI 0.28-0.71, p<0.001), and there was no impact on toxicity or quality of life. With five years of follow-up, there was no difference in prostate cancer-specific survival nor overall survival for now, but this might become significant with longer follow-up.

Proton beam technology has the physical advantage of depositing energy in the tissue at the end-of-range, thus potentially sparing critical normal tissues such as the rectum and bladder in prostate cancer patients (82). Studies to date have not demonstrated an improvement in toxicity rates or clear benefit for protons. For example, a recent multi-institutional analysis of 1850 early-stage prostate cancer patients treated with either moderately hypofractionated photon or proton therapy on a registry demonstrated low rates of toxicity and no difference in late gastrointestinal or genitourinary toxicity (83). Yet, there are several ongoing trials evaluating protons versus photons for localized prostate cancer that will help to guide our understanding of the potential benefit for protons in this clinical space. A large ongoing randomized phase III trial of proton therapy versus intensity-modulated radiation therapy for low- to intermediate-risk prostate cancer called Prostate Advanced Radiation Technologies Investigating Quality of Life, or PARTIQoL (NCT01617161), as well as large prospective observational cohorts such as a Prospective Comparative Study of Outcomes with Proton and Photon Radiation in Prostate Cancer (COMPPARE, NCT03561220) and the Japanese multi-institutional prospective registry (UMIN000025453), will help to inform the debate between protons versus photons for localized prostate cancer.

**Figure 1:**
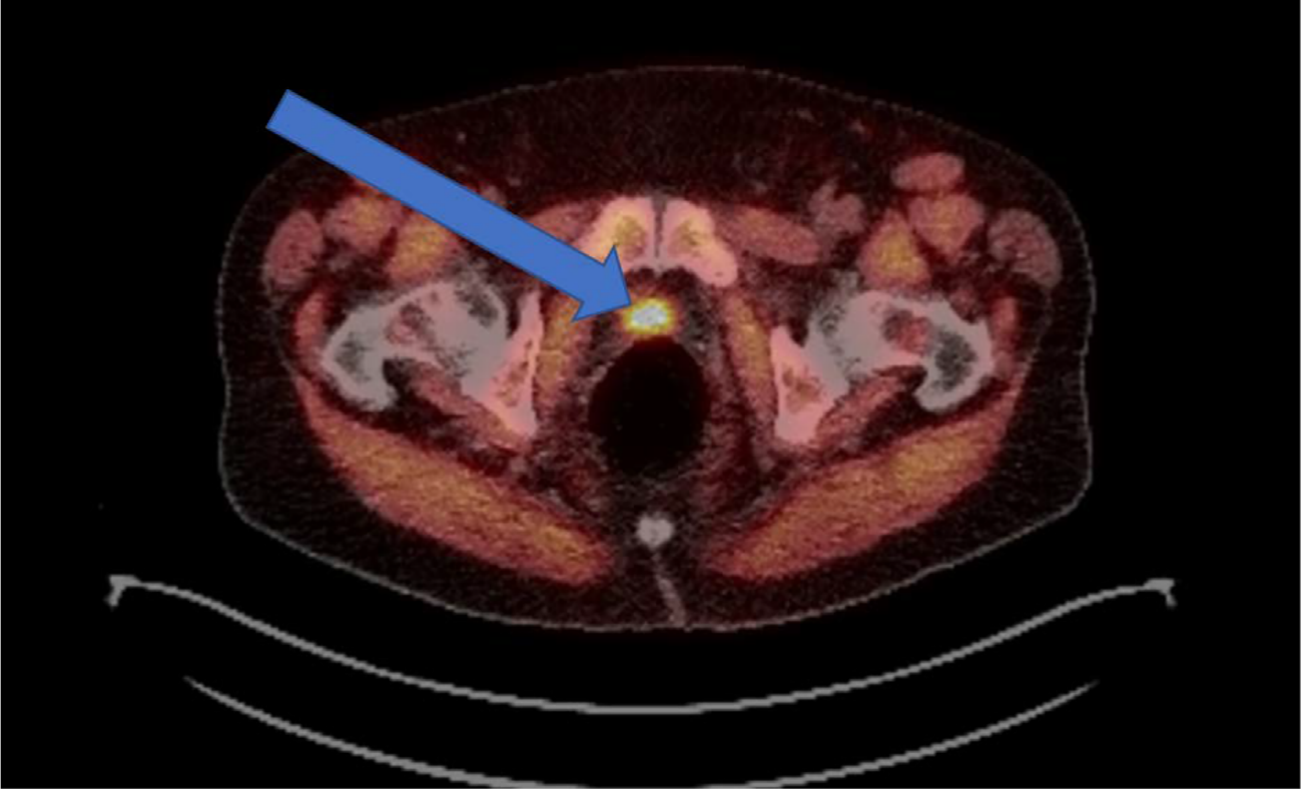
Example of an ^18^F-fluciclovine PET avid lesion in a biochemically recurrent prostate cancer patient. Demonstration of a 2.3-cm ^18^F-fluciclovine PET-avid lesion in the prostatectomy bed. Patient was post-radical prostatectomy and presented with a PSA of 0.84 ng/mL.

Novel imaging techniques surrounding the identification and detection of prostate cancer as discussed above are changing how to treat this disease with radiation therapy, particularly in the post-operative setting. The role of PET/CT imaging was previously limited, however the introduction of novel imaging tracers including ^18^F-fluciclovine PET (**Figure 1**) and prostate-specific membrane antigen (PSMA) targeted agents has the potential to change clinical practice. Studies suggest that these novel radiotracers can modify radiation treatment intensification (84) as well as lead to an early improvement in failure rates (85). The LOCATE trial demonstrated increased detection of 1 or more recurrences using ^18^F-fluciclovine PET/CT in men with biochemical recurrence (122 of 213 patients, 57%), and 59% of patients had a change in management after the scan (86). Similarly, the FALCON trial demonstrated that the use of ^18^F-fluciclovine PET/CT in 104 men with biochemical recurrence resulted in 64% of patients with a change in treatment management (87). Multiple prospective trials are underway in various prostate cancer settings (diagnostic, localized, post-operative, recurrent, metastatic) to further standardize and validate its use in various clinical settings.

As precision oncology continues to evolve for the management of prostate cancer with improved biomarkers and improved detection of disease, the role of radiation therapy is also evolving, particularly with regards to the definitive management of oligometastatic disease. Oligometastatic disease refers to a stage where metastatic disease is still limited, and aggressive therapy directed at involved lesions may improve outcomes. The definition of oligometastatic prostate cancer varies, as the CHAARTED study defined oligometastatic disease as ≤3 metastases, and no visceral metastases (88), yet other studies define oligometastatic disease as ≤5 metastases (89). In this space with evidence of overall limited disease, stereotactic ablative radiation therapy (SABR) may become a part of regular treatment management.

Both the HORRAD (90) and STAMPEDE (91) trials evaluated the role of definitive local treatment to the primary prostate in the setting of metastatic disease. In both trials, there was no difference in overall survival with the use of prostate primary-directed radiation therapy. However, on subgroup analysis in HORRAD, there was a trend toward benefit in overall survival with radiation therapy in men with low metastatic burden (defined as ≤5 metastases, HR 0.68, 95% CI 0.42-1.10). Based on this, analysis by metastatic burden was a prespecified subgroup analysis in STAMPEDE. The subgroup analysis met many of the subgroup analysis criteria put forth by Sun et al. (92). Aa survival benefit was found in favor of primary prostate radiotherapy in men with ≤3 metastases (HR 0.68, 95% CI 0.52-0.90, p=0.0098). A secondary analysis of this trial demonstrated a significant survival benefit in patients with lymph node only metastases (HR 0.60, 95% CI 0.33-1.09) as well as a failure-free survival benefit beyond 4 bone metastases up to 8 bone metastases (93). Overall, these are encouraging results but need to be further evaluated prospectively, particularly with new imaging modalities.

With respect to treatment of the metastatic lesions rather than the primary, the STOMP trial evaluated the benefit of metastasis-directed therapy (MDT, either surgery or radiation) in patients with biochemical recurrence after primary prostate treatment and ≤3 metastases, with a primary endpoint of ADT-free survival (94). After a median follow-up of 3 years, aggressive metastasis-directed therapy did increase ADT-free survival (median ADT-free survival was 13 months [80% CI 12-17 months] for surveillance versus 21 months [80% CI 14-29 months] in MDT group, p=0.11). Separately, the ORIOLE trial, a randomized phase 2 trial, evaluated observation versus SABR to metastatic disease in men with 1-3 metastases, found that, with a median follow-up of 18.8 months, SABR improved median progression-free survival (PFS, not reached versus 5.8 months, HR 0.30, 95% CI 0.11-0.81, p=0.0023) (95). This trial incorporated the use of PSMA-PET, thus this is a contemporary evaluation of SABR in the oligometastatic disease space. More work needs to be performed to further define oligometastatic disease (number of metastases, oligoprogressive versus oligorecurrent, etc), understand the benefit of treatment to the primary versus metastases (versus both), benefit in setting of standard and escalated therapies, and others, but there remain many exciting opportunities for exploration into these questions to define the role of radiation therapy in this space.

## Bladder Cancer

There are two standard treatment options for muscle-invasive bladder cancer: 1) radical cystectomy, and 2) bladder preservation therapy (or trimodality therapy, TMT). Bladder preservation therapy is comprised of a combination of maximal transurethral resection of bladder tumor (TURBT), followed by chemoradiation. Molecular understanding of individual muscle-invasive bladder tumors may lead to predictive and prognostic biomarkers that can aid with treatment selection for individuals. Already, there are several promising candidates (96).

Bladder tumors frequently display mutations in DNA repair pathways, which likely drive bladder tumor development (97). MRE11 has demonstrated promise as a biomarker of radiation response. One study evaluated immunohistochemical staining of MRE11 in a cohort of patients treated with radiation alone (98). It was determined that patients with the lowest amounts of MRE11 staining had an associated worse 3-year cancer-specific survival. This was validated in a study evaluating MRE11 expression in tissues from 6 NRG/RTOG bladder-sparing radiation protocols. Low levels of MRE11 nuclear/cytoplasmic expression scores were associated with significantly higher disease-specific mortality (99). Other groups have demonstrated a similar association. Laurberg et al. demonstrated low MRE11 staining was associated with worse disease-specific survival in a cohort of 148 patients treated with bladder preservation (100). They also found no associated with MRE11 staining and outcomes among patients who were treated with cystectomy. In a study by Teo et al., a single-nucleotide polymorphism (SNP) in the *MRE11A* gene was associated with worse outcomes among patients treated with radiation therapy, but not among patients treated with cystectomy (101). Interestingly, this SNP was not associated with increased or decreased MRE11 measured by immunohistochemistry.

Further investigations into DNA repair pathway alterations have been performed more often in cohorts of those who received neoadjuvant chemotherapy followed by cystectomy or in those with metastatic disease. However, a small study of 48 patients treated with bladder preservation found deleterious mutations in DNA repair pathways, in particular, *ERCC2*, were associated with improved outcomes after chemoradiation (102). More work needs to be done in bladder preservation-specific cohorts.

Separately, alterations in signal transduction pathways have also been implicated in bladder preservation. In a study using patients enrolled in four prospective bladder preservation studies (RTOG 8802, 8903, 9506, 9706), EGFR expression assessed by immunohistochemistry was associated with improved outcomes in both univariate and multivariate analyses. Conversely, HER2 expression *via* immunohistochemistry was associated with poorer outcomes, specifically with reduction in complete response after chemoradiation (103). This latter finding was confirmed by Inoue et al. in a cohort of 119 patients treated with bladder preservation therapy (104). HER2 overexpression was associated with pathologic incomplete response and worse cancer-specific survival, suggesting resistance to chemoradiation. The RTOG 0524 phase I/II trial evaluated the use of trastuzumab in patients who were HER2/neu 2+ or 3+ along with concurrent paclitaxel and radiation, versus radiation and concurrent paclitaxel in patients who were HER2/neu-negative or 1+ by immunohistochemistry (105). It was found that both groups had similar complete response rates, thus suggesting that in patients with HER2/neu 2+ or 3+ expressing tumors, the addition of trastuzumab mitigated the previously associated worse prognosis. This finding needs to be further evaluated in a randomized study but demonstrates the ability of biomarker-driven trials to improve outcomes in challenging diseases.

There has been work defining various molecular subtypes based on gene expression profiles (106–110). The subtypes, broadly characterized based on luminal and basal gene expression patterns, have been correlated with response to treatments including cystectomy and neoadjuvant chemotherapy. However, their association with response to chemoradiation is not clear. In one of the largest studies of molecular subtypes within a cohort of patients receiving bladder preservation therapy, specifically TMT, four subtypes were described, luminal, luminal-infiltrated, basal, and claudin-low (111). There was no association with complete response, disease-specific survival or overall survival within the cohort. Further investigation and validation with other TMT/bladder preservation cohorts is necessary.

In patients who are not eligible for concurrent chemotherapy, the use of carbogen and nicotinamide to modify hypoxia in tumors resulted in improved survival compared to radiation alone in the BCON trial (112). Upon further analysis by molecular subtype, patients with a basal subtype had greater benefit with hypoxia modification while those with a luminal subtype had no benefit (113). A 24-gene hypoxia signature was developed and validated in the BCON cohort and found that patients with “high-hypoxia” per the signature had improved outcomes compared to those with “low-hypoxia” with the use of hypoxia modification (114). Both the hypoxia signature as well as the molecular subtype have yet to be validated prospectively to guide use of hypoxia modification but serve as early tools to aid in development of future trials.

Finally, identifying biomarkers related to immune checkpoint inhibition (ICI) response in muscle-invasive bladder cancer is of critical importance, particularly given the potential for improved response when combining ICIs with radiation therapy. There are multiple ongoing clinical investigations into the potential synergy of combination ICI + radiation therapy in this patient population. The aforementioned work evaluating molecular subtypes within a cohort of patients receiving TMT also evaluated immune signatures based on gene expression, finding that signatures associated with T-cell activation and interferon-gamma signaling were associated with improved disease-specific survival in the TMT cohort (111). In a comparison cohort of patients with muscle-invasive bladder cancer treated with neoadjuvant chemotherapy and radical cystectomy, this association did not hold. These promising data demonstrate that immune-related biomarkers may have implications for TMT in muscle-invasive bladder cancer, and potentially the combination of TMT + ICIs. This will need to be further examined in the ongoing trials evaluating TMT + ICI, such as the INTACT: SWOG/NRG 1806 study, evaluating chemoradiotherapy +/- atezolizumab in muscle-invasive bladder cancer (NCT03775265); the KEYNOTE-992 study, evaluating chemoradiotherapy +/- pembrolizumab in muscle-invasive bladder cancer (NCT04241185); the CCTG BL13 trial, evaluating chemoradiotherapy followed by +/- adjuvant durvalumab; and the INSPIRE: ECOG-ACRIN/NRG EA8185 trial, evaluating chemoradiation +/- durvalumab in node-positive urothelial carcinoma (NCT04216290).

Liquid biopsy tools such as CTCs and ctDNA are similarly being investigated as prognostic biomarkers in bladder cancer as they are in prostate cancer (115, 116). CTCs and ctDNA will be collected and assessed in both the abovementioned INTACT and INSPIRE trials to determine their role as predictive biomarkers for overall outcomes after bladder preservation therapy. The presence of circulating biomarkers is also being explored in the surveillance setting post-treatment. One study in patients undergoing neoadjuvant chemotherapy followed by radical cystectomy found that the presence of ctDNA was prognostic for worse outcomes overall (115). This has not yet been evaluated in a TMT cohort but the data from INTACT and INSPIRE will help elucidate the role of liquid biomarkers in TMT-specific cohorts. 

### Advances in Radiotherapy Techniques, Treatment Delivery

Advances in image-guidance for radiation therapy has facilitated both dose escalation, hypofractionation, and adaptive planning for bladder cancer patients. Older trials evaluated multiple options for radiation dose, fields, and frequency of radiation treatment, thus there is no standard at this time. The INTACT trial is very inclusive and allows a variety of radiation fields, per physician discretion. Regarding dose, a recent meta-analysis of two randomized, controlled, phase 3 trials in the UK demonstrated that a hypofractionated schedule of 55 Gy in 20 fractions is non-inferior to conventional fractionation (64 Gy in 32 fractions) (117). However, there appears to be a non-trivial increase in unacceptable gastrointestinal grade 3 toxicity when using hypofractionation in combination with immune checkpoint inhibitors, based on results from a phase I trial of atezolizumab and chemoradiation (50 Gy/20 fractions) for muscle-invasive bladder cancer (118) as well as a phase I trial of pembrolizumab and weekly radiation of 6 Gy per fraction to a dose of 36 Gy (119). Both trials had a small number of patients (n=8 and 5 respectively), and both were stopped early due to the dose-limiting toxicities observed. Thus, at this time, the INTACT trial uses conventional fractionation to avoid events that may contribute to dose-limiting toxicities.

To better delineate the primary bladder tumor, other imaging modalities are being explored that may aid in tumor-directed treatment. FDG-PET/CT may improve initial staging to better select patients for TMT, but physiologic uptake in the bladder limits its ability to better delineate the bladder tumor (120, 121). Multiparametric MRI is being explored to improve bladder tumor staging with advanced identification of muscle-invasion. MRI may also improve response assessment after bladder preservation therapy (122–125). At this time, further work is necessary to define the role of multiparametric MRI in the management of muscle-invasive bladder cancer.

Ongoing trials are evaluating the utility of adaptive planning for treatment of advanced bladder cancer. One such trial is the RAIDER study (NCT02447549), which is a randomized phase II trial of either standard planning and radiation delivery, adaptive image guided tumor-focused radiation, or adaptive image guided dose-escalated tumor boost radiation. The primary endpoint is the proportion of patients meeting radiation dose constraints to the bladder, bowel, and rectum in the dose-escalated group, as well as the proportion of patients experiencing severe late side effects following treatment.

In the field of precision oncology, there are many exciting opportunities for radiation in the treatment of muscle-invasive bladder cancer. As ongoing trials start to close and more study into potential biomarkers is completed, the resulting data will aid in our improved selection and treatment of candidates for bladder-preservation.

## Testicular Cancer

In testicular seminoma, current treatment approaches have made this disease highly curable. Historically, radiation was the primary treatment for this disease, but the preferred treatment landscape has changed. For stage I seminoma, active surveillance is now the preferred treatment option (126). Emphasis on biomarkers of recurrence is necessary, as there are no current clinicopathologic variables that can be relied upon. Serum tumor markers are rarely elevated in a recurrence setting, and multiple studies suggest that they are unnecessary during surveillance follow-up (127, 128). Tumor size has been suggested as a risk factor for recurrence, however data is mixed on its prognostic ability (129, 130). miRNAs have demonstrated early promise as both diagnostic and prognostic markers (131, 132) but validation is required. The surveillance strategy consists of frequent computed tomography scans and follow-up. Yet, the seminoma population is very young, thus there is an emphasis to minimize irradiation. The Trial of Imaging and Surveillance in Seminoma Testis (TRISST, NCT00589537) evaluated the utility of decreased number of scans (from 7 to 3) as well as replacing CT scans with MRI (133). Results were recently presented at the 2021 GU Cancers Symposium and found that MRI is non-inferior to CT, and thus should be recommended, and a 3-scan schedule is non-inferior to 7 scans. The surveillance paradigm will likely shift given these recent findings, and this trial reaffirms that surveillance is both safe and effective in stage I seminoma.

For early stage II disease (specifically stage IIA), treatment options include either radiation therapy or chemotherapy (typically 3-4 cycles of etoposide/cisplatin/bleomycin). Radiation therapy is preferred over chemotherapy given the favorable tolerability and toxicity profile (126). However, greater precision is needed with selection of treatment. There are currently no tools to help inform the decision between radiation or chemotherapy in this clinical setting.

When radiation therapy is indicated, there have been efforts to further limit radiation dose to organs-at-risk in this young population. Originally, radiation was delivered using 30 Gy in 15 fractions in the adjuvant setting. Yet, the recognition of seminoma as highly radiosensitive led to the pivotal trial exploring 30 Gy/15 fractions versus 20 Gy in 10 fractions (134). After a median follow-up of 61 months, it was determined that 20 Gy was just as effective and non-inferior to 30 Gy. Further reduction in dose to organs-at-risk may be accomplished using proton beam therapy. Proton beam technology is a promising treatment modality for this patient population given its unique physical characteristics. A recent study comparing patients between proton beam therapy and photon-based treatment demonstrated excellent outcomes and no in-field secondary malignancies (135), although this data is limited with only 55 patients included and a median follow-up of 61 months. Separately, a dosimetric modeling study demonstrated superior sparing of organs-at-risk with protons as compared to photons. Proton beam therapy was estimated to avert 300 excess second cancers among 10,000 men treated at a median age of 39 and surviving to age 75 (136). Proton beam therapy should be strongly considered and further evaluated for men with testicular seminoma.

Decreased field size has been highlighted specifically to further limit dose to organs-at-risk. Emphasis on decreasing field size was evaluated in a trial for stage I testicular seminoma patients, randomizing patients to either a para-aortic strip or ipsilateral iliac lymph node irradiation (dog-leg field) (137). After a median follow-up of 4.5 years, the para-aortic strip was non-inferior to the dog-leg field and reduced toxicity; it is now accepted as standard-of-care for adjuvant radiation treatment for stage I seminoma. More recently, an analysis of metastatic lymph node positives respective to vascular anatomy was performed in seminoma patients and suggested modified treatment fields based on vascular anatomy to decrease normal tissue irradiation (138). This study demonstrated that the superior border of the treatment field can safely be decreased from the T10/T11 interspace to the T11/T12 interspace. In addition, this has led to a greater emphasis on tailored nodal treatment fields based on vascular, rather than bony, anatomy.

Overall, more work is needed in the field of biomarkers for testicular cancer, particularly as it relates to radiotherapy, in the surveillance setting, for treatment selection and for response to treatment.

## Renal Cell Carcinoma

The management of renal cell carcinoma (RCC) has been revolutionized by targeted kinase inhibitors (TKIs) as well as immunotherapy. Traditionally, RCC was deemed “radioresistant” and the role for radiation therapy was limited to mostly palliation. However, the rapid advancement of on-treatment image guidance, as well as highly conformal techniques to deliver a high-dose-per-fraction, has paved the way for stereotactic ablative radiation therapy (SABR) to play a role in definitive treatment of RCC (139–141). A 2019 meta-analysis of 26 studies targeting primary RCC with SABR demonstrated excellent local control and low grade 3-4 toxicity rates (142). Regarding kidney function, a prior study of 21 patients with inoperable RCC demonstrated reasonable change in mean GFR at 2 weeks (+0.6 +/- 11.3 ml/min), 3 months (+3.2 +/- 14.5 ml/min), and 1 year (-8.7 +/-13.4 ml/min) (143). Ongoing trials are further evaluating the safety and efficacy of SABR to primary RCC (NCT02853162, NCT03108703, NCT01890590, NCT02613819, NCT03747133) and will help to establish the role of SABR for primary RCC.

Separately, there are numerous studies demonstrating a potential synergistic antitumor effect with SABR in combination with targeted therapies for metastatic RCC (mRCC). For example, SABR to an “oligoprogressive” lesion was found to extend the efficacy of sunitinib from 14 to 22 days (141). There is a lot of interest and ongoing trials evaluating the efficacy of combined immunotherapy with radiation, given case reports that have described an observed abscopal effect in the setting of both radiation and immune checkpoint inhibition (144). The phase II NIVES study (NCT03469713) is a single-arm study, evaluating the role of SABR to metastatic lesions in mRCC patients who receive nivolumab. Early data demonstrate a median PFS of 4 months, which is not much different from the nivolumab alone arm on CheckMate025, a trial randomizing mRCC patients to nivolumab versus everolimus (145). The RADVAX trial (NCT03065179) is a single-arm study evaluating the role of SABR to metastases in mRCC patients who receive both nivolumab and ipilumumab, with a median PFS of 8.2 months thus far. This is also not much different from the nivolumab + ipilumumab arm in CheckMate214, which randomized mRCC patients to either dual checkpoint inhibition or sunitinib (146). However, in the RAPPORT trial that was presented at the recent 2021 GU Cancers Symposium, patients with low burden of metastases received SABR (20 Gy x 1) and pembrolizumab (147). The treatment was well tolerated and the median PFS was 15.6 months, which is improved over the KEYNOTE-427 trial of pembrolizumab monotherapy (PFS of 7.1months). Further work is necessary to understand appropriate patient selection to a confer a benefit for SABR. The CYTOSHRINK trial is a phase II trial of nivolumab and ipilumumab +/- SABR to the primary RCC in mRCC patients (NCT04090710). Other trials are being opened in this space to evaluate the role of SABR to the primary or the primary + metastases in combination with immune checkpoint inhibitors to potentiate the effect of immunotherapy and improve outcomes in this disease space.

## Summary

Advances in technology have led to a greater understanding of the molecular characterization of genitourinary cancers. Separately, developments in radiation therapy have led to improved tumor targeting as well as decreased dose to surrounding normal tissues. However, there is an urgent need to incorporate molecular information about various genitourinary malignancies to personalize radiation treatment. Just in the past few years, considerable progress has been made within the GU field with many promising biomarkers that have the potential to optimize radiation management that need to be validated. There remain many exciting opportunities for biomarker discovery as well as a need to validate the utility of biomarkers into initial management of genitourinary malignancies. We advocate for the incorporation of known tumor biomarkers into prospective clinical trials as well as for incorporation of translational studies for further biomarker discovery. Continued effort is necessary to one day fully integrate tumor biology to inform management decisions, with the ultimate goal of improving outcomes for our patients. 

## Author Contributions

Concept and design: SK and JE. Drafting of the manuscript: SK and JE. Critical revision of the manuscript for important intellectual content: SK and JE. All authors contributed to the article and approved the submitted version.

## Conflict of Interest

JAE has served as consultant to: Blue Earth Diagnostics, Boston Scientific, AstraZeneca; and on advisory boards for Myovant Sciences, Roivant Pharma, Merck and Progenics.

The remaining author declares that the research was conducted in the absence of any commercial or financial relationships that could be construed as a potential conflict of interest.
